# Transcriptional response of *Escherichia coli* to ammonia and glucose fluctuations

**DOI:** 10.1111/1751-7915.12713

**Published:** 2017-04-26

**Authors:** Joana Danica Simen, Michael Löffler, Günter Jäger, Karin Schäferhoff, Andreas Freund, Jakob Matthes, Jan Müller, Ralf Takors, RecogNice‐Team, Ronny Feuer, Joachim von Wulffen, Julia Lischke, Michael Ederer, David Knies, Samantha Kunz, Oliver Sawodny, Olaf Riess, Georg Sprenger, Natalie Trachtmann, Alexander Nieß, Alexander Broicher

**Affiliations:** ^1^ Institute of Biochemical EngineeringUniversity of StuttgartAllmandring 3170569StuttgartGermany; ^2^ Institute of Medical Genetics and Applied GenomicsUniversity of TübingenCalwerstr. 772076TübingenGermany

## Abstract

In large‐scale production processes, metabolic control is typically achieved by limited supply of essential nutrients such as glucose or ammonia. With increasing bioreactor dimensions, microbial producers such as *Escherichia coli* are exposed to changing substrate availabilities due to limited mixing. In turn, cells sense and respond to these dynamic conditions leading to frequent activation of their regulatory programmes. Previously, we characterized short‐ and long‐term strategies of cells to adapt to glucose fluctuations. Here, we focused on fluctuating ammonia supply while studying a continuously running two‐compartment bioreactor system comprising a stirred tank reactor (STR) and a plug‐flow reactor (PFR). The alarmone ppGpp rapidly accumulated in PFR, initiating considerable transcriptional responses after 70 s. About 400 genes were repeatedly switched on/off when *E. coli* returned to the STR. *E. coli* revealed highly diverging long‐term transcriptional responses in ammonia compared to glucose fluctuations. In contrast, the induction of stringent regulation was a common feature of both short‐term responses. Cellular ATP demands for coping with fluctuating ammonia supply were found to increase maintenance by 15%. The identification of genes contributing to the increased ATP demand together with the elucidation of regulatory mechanisms may help to create robust cells and processes for large‐scale application.

## Introduction

Aerobic, large‐scale production processes are bound by technical limits such as maximum oxygen transfer rate (typically 150–180 mmol l^−1^ h^−1^) or cooling capacity. Basically, such limits mirror the design compromise between technical feasibility and economic constraints. Consequently, the metabolic activity of the producer cells needs to slow down during the production phase to stay within given borders. This necessary control of metabolic activity is typically achieved by limiting nutrient supply. Besides carbon limitation (e.g. glucose), nitrogen limitation (e.g. ammonia) is often used to control the fermentation process. It has been found that nitrogen limitation even increased cell specific glucose uptake rates (Hua *et al*., 2004) which is highly desirable in microbial production processes.

However, as bioreactor dimensions increase, spatial inhomogeneities (e.g. in nutrients, dissolved gases and pH) arise in the cellular microenvironment mainly due to insufficient mixing caused by limited power input (Bylund *et al*., 1998; Humphrey, 1998; Lapin *et al*., 2006; Takors, 2012). The nutrient gradients are frequently crossed by the cultured cells, thereby triggering intracellular changes in response to the changes in external nutrients.

Our knowledge concerning the fundamental regulatory processes that enable *Escherichia coli* to successfully adapt to changing nutrient availabilities is already profound (Hua *et al*., 2004; Traxler *et al*., 2008; Shimada *et al*., 2011; You *et al*., 2013; Wulffen *et al*., 2016). However, such studies have been performed in ‘well‐mixed’ systems that do not mirror well the fluctuating environment of large‐scale bioreactors. Examples revealing negative effects of substrate gradients (e.g. lowering of biomass yield and by‐product formation) on large‐scale process performance are rare (Larsson *et al*., 1996; Bylund *et al*., 1998), not taking into account the impact of fluctuating nitrogen supply. Ammonia and glucose are both essential nutrients whose extracellular availability is crucial for the coordinated allocation of intracellular resources that eventually lead to formation of biomass and product (Doucette *et al*., 2011; You *et al*., 2013). Therefore, this report aims at elucidating the transcriptional responses of *E. coli* under fluctuating ammonia availability.

Today, the so‐called scale‐down devices, which simulate large‐scale gradients, are often used to investigate both cellular response and sensitivity at the transcriptional (Schweder *et al*., 1999; Lara *et al*., 2006; Buchholz *et al*., 2014; Löffler *et al*., 2016), proteomic (Limberg *et al*., 2016) and product level (Neubauer *et al*., 1995; Lemoine *et al*., 2015). Usually, batch or fed‐batch cultivations are performed with and without imposing gradients to identify phenotypic differences in the fermentations. Such approaches possess the inherent drawback that process dynamics superimposed on gradient‐induced regulation dynamics make it difficult to decipher the latter independent of the former. Alternatively, we have presented a continuous cultivation approach consisting of a stirred tank reactor (STR) coupled to a plug‐flow reactor (PFR; Löffler *et al*., 2016). In this way, a steady‐state reference state without gradients and a subsequent period with gradients can be investigated separately and the two states compared. Using this approach, the tactical (short‐term) and the strategic (long‐term) response of wild‐type *E. coli* to periodically changing glucose availability could be deciphered. Based on these results, strategies to engineer *E. coli* strains more suitable for large‐scale production were suggested, including identifying candidates for gene deletion that could help to minimize unwanted ATP loss caused by the periodic switching on and off of genes.

Similar experiments were performed in this study with a focus on ammonia availability instead of glucose. The aim of the study was then to investigate short‐ and long‐term responses to a fluctuating ammonia supply, while mimicking large‐scale bioreactor conditions. The data obtained were compared with previously reported glucose induced transcriptional dynamics (Löffler *et al*., 2016) to elucidate substrate‐related similarities and differences. The results should allow an understanding of how *E. coli* adapts to changes in the environment of a bioreactor; it is promising that this will support future bacterial strain engineering and bioreactor design for large‐scale applications.

## Results

### Experimental design of the periodic stimulation studies

**Figure 1:**
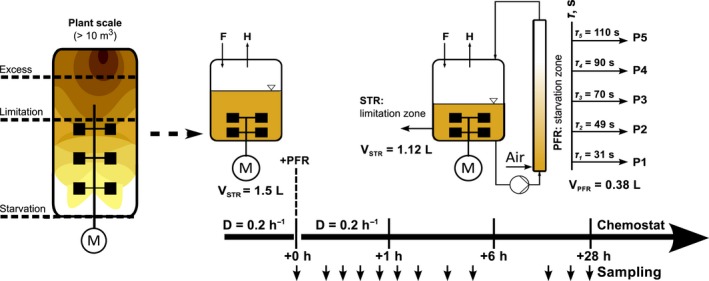
Experimental design of the two‐compartment system. The two‐compartment device consists of a stirred tank reactor (STR) connected to a plug‐flow reactor (PFR). The STR‐PFR was designed to give a simplified representation of some of the periodic substrate variations experienced by cells in large‐scale bioreactors: the limiting substrate is fed into the well‐mixed STR (limitation zone) and residual substrate is quickly consumed when microbial cells enter the PFR, leading to the development of a starvation zone. A continuous process strategy was chosen to maintain a constant volume and average dilution rate of *D* = 0.2 h^−1^ in the system. The steady state prior to PFR onset at time zero was used as the reference state (S_0_). Samples were taken at eleven distinct time points over 28 h. The system is equipped with five PFR sample ports (P1‐5) at defined residence times τ (s), as well as STR sample port S. The residence times in the connecting loops from the STR outlet to P1 and P5 to the PFR outlet were 31 and 15 s respectively. This results in a total mean PFR residence time of τ_PFR_ = 125 s (for calculations see Methods S1). F = feed; H = harvest.

The cellular response to fluctuating ammonia availability was examined using the STR‐PFR two‐compartment system previously described in Löffler *et al*. (2016) using similar operating conditions. In the STR, cells are grown under conditions of limited ammonia from which a fraction is then continuously drained into the PFR loop. In the PFR, cells shift from ammonia limitation to ammonia starvation mode as they consume and exhaust any residual ammonia before they return to the STR. Five sample ports (P1–P5) were located along the PFR to monitor the short‐term cellular response, while long‐term effects were followed via sampling from the STR sample port S (Fig. 1). The set‐up was characterized using tracer experiments to confirm plug‐flow behaviour in the PFR (Bodenstein number *Bo* = 84) and to determine the average residence time $\overset{¯}{\tau }$ of the cells in each compartment ($\bar{\tau_{\mathrm{STR}}}$ = 6.2 min and $\bar{\tau_{\mathrm{PFR}}}$ = 125 s) (for details, see Methods S1 and Löffler *et al*., 2016). Figure 1 further depicts the individual residence times determined for each PFR sample port. The PFR‐to‐STR volume ratio of 1:3 was designed based on the results of Lapin *et al*. (2006) who simulated the growth performance of *E. coli* under ideally and poorly mixed fed‐batch conditions. A recent study indicates that large‐scale starvation zones might even encompass 50% of the total reactor volume (Haringa *et al*., 2016). Hence, the experimental setting in our study still agrees with large‐scale recirculation times (Junker, 2004; Noorman, 2011) and the configurations used in other scale‐down studies (Schweder *et al*., 1999; Amanullah *et al*., 2001).

As first described in Löffler *et al*. (2016), the STR‐PFR system was operated in continuous mode (Novick and Szilard, 1950) with ammonia as the growth‐limiting substrate which was fed continuously into the STR. To achieve this, we established steady‐state conditions in the STR prior to and during connection of the PFR cell recycle loop. In this way, a distinct reference state was established. By contrast, in conventional scale‐down set‐ups conducted in fed‐batch mode, the overall effects resulting from changing process conditions and/or various external stimuli (e.g. changes in residence time distribution) often make an appropriate interpretation of observations difficult. The two‐step cultivation process is illustrated in Fig. 1. First, the reference steady state (S_0_) with a growth rate of 0.2 h^−1^ was established in the STR. The exemplary dilution rate of 0.2 h^−1^ was chosen to allow comparability to previously performed glucose experiments (Löffler *et al*., 2016). Moreover, it met the demand of comprehensively studying fluctuations between ammonia limitation and depletion, while mimicking large‐scale conditions. However, the system is not restricted to these conditions as smaller (or higher) growth rates may be installed accordingly providing the possibility to study a multitude of physiological responses. To characterize the intrinsic steady‐state fluctuations, S_0_ was sampled three times during a 16 h period following establishment of the steady state. Then, the PFR was connected and the culture was thoroughly characterized by measuring key process parameters (biomass, glucose, ammonia, oxygen and carbon dioxide), the intracellular level of ppGpp and the transcriptome (RNA‐sequencing analysis). Using the observed ammonia uptake rate of 56 μg l^−1^ s^−1^ and assuming a detection limit of 2.5 mg_am_ l^−1^ as the residual amount that entered the loop, ammonia should be fully depleted after 45 s. Consequently, the PFR can be divided into a growth zone with ammonia (bordered by a 45 s residence time) and a non‐growth zone without ammonia. Because the average growth rate of the total STR‐PFR remained at 0.2 h^−1^, the effective growth in the ammonia containing compartment was μ_STR_ = 0.24 h^−1^ (for details, see also Löffler *et al*., 2016). The STR‐PFR cultivation was performed twice under identical experimental conditions.

### Short‐term response to ammonia shortage

**Figure 2:**
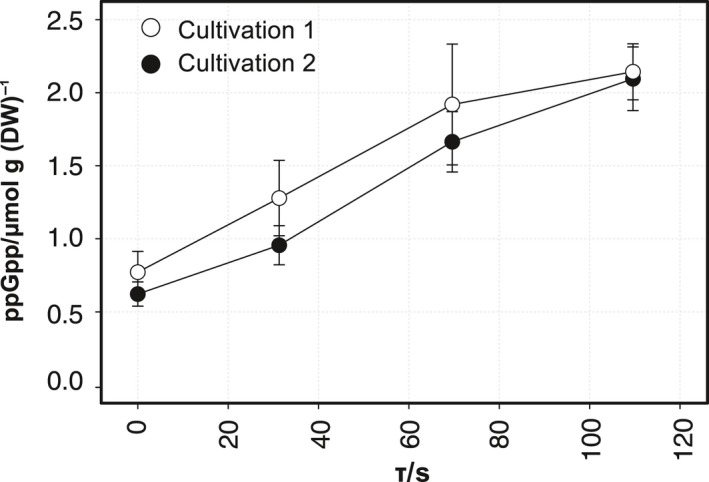
Short‐term accumulation of ppGpp over residence time in the PFR. Samples from two independent STR‐PFR cultivations were analysed at different process time points. Time profiles show the average ppGpp concentration in μmol g (DW)^−1^ at process times of 25 min, 120 min and 28 h (mean ± SD).

Intracellular levels of the alarmone ppGpp accumulated along the PFR with an average increase of 3.5‐fold (Fig. 2, Fig. S1). The level nearly doubled after only 30 s of ammonia shortage. The small variance bars indicate that the response was highly repeatable, showing similar patterns irrespective of the process time.

**Figure 3:**
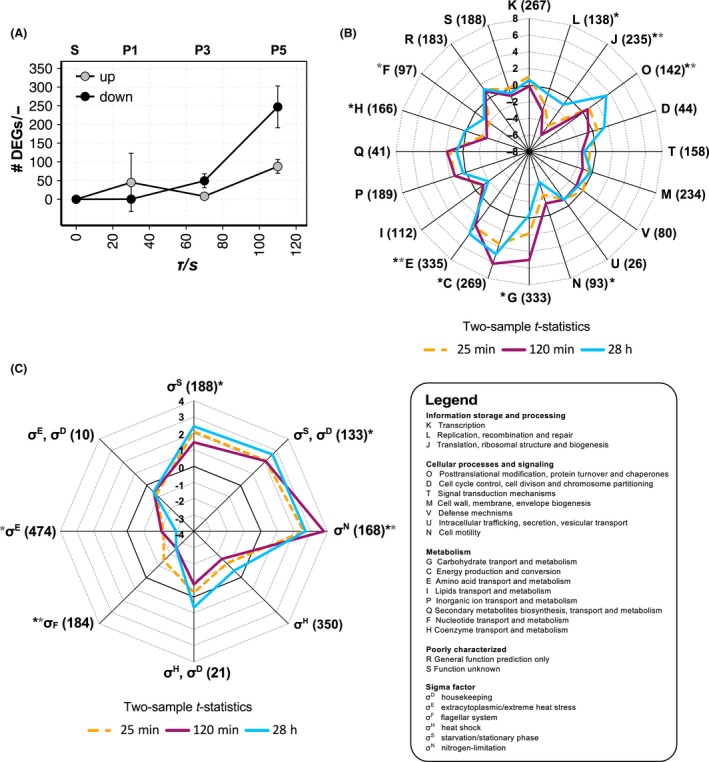
Transcriptional responses to short‐term nitrogen shortage. A. Number of DEGs whose expression was increased (grey circles) or reduced (black circles) between PFR and STR. Time courses are shown as mean ± SD calculated from samples withdrawn at 25 min, 120 min and 28 h after PFR onset. DEGs are defined as having an FDR < 0.01 and log_2_ fold change ≥ |0.58|. B. COG functional categories (Tatusov *et al*., 2000) and (C) sigma factor regulation (Salgado *et al*., 2013) pattern for the comparison of PFR sample port P5 vs. S, visualized as spider graphs. Because no COG or sigma factor annotation was found for 559 and 428 of 3889 genes, respectively, these genes were excluded from the statistical analysis. The *t*‐statistics pattern from GAGE (Luo *et al*., 2009) is shown for three representative time points: 25 min (gold dotted line), 120 min (magenta line) and 28 h (blue line) after the PFR was coupled to the STR. Sets including less than 10, or above 500, genes were omitted from the analysis. Functional groups that were significantly changed with an FDR < 0.05 at a minimum of one time point using either GAGE (black asterisk) or hypergeometric distribution (grey asterisk) analysis are indicated. Overlapping sigma factor sensitivities: gene regulation may occur by each of the sigma factors depicted because multiple promoters exert control.

Analogous to the study conducted by Löffler *et al*. (2016), RNA‐sequencing data were obtained. After exclusion of very poorly expressed genes by primary filtering, 3889 genes remained for analysis. The fast transcriptional response to ammonia shortage was determined by comparing PFR samples with STR equivalents taken at the same process times. About half of the genes with a false discovery rate (FDR) < 0.01 showed fold changes below 1.5. The number of differentially expressed genes (DEGs) along the PFR with fold changes ≥ 1.5 is shown in Fig. 3A. Within 110 s, we found DEGs with log_2_ fold changes ranging from between −3.8 and 3.2 corresponding to 14‐fold down‐ and ninefold upregulation respectively. During the first 70 s, 40 and 78 genes were up‐ or downregulated at least 1.5‐fold respectively. Subsequently, the number of up‐ and downregulated DEGs at the PFR outlet quickly increased to 57–112 and 165–384 respectively. Analogous to the ppGpp profiles, the DEG profiles revealed high reproducibility over process time (Fig. 3A). Only P1 values showed relatively high variance as the data set at 120 min encompassed unique expression events that could not be fully explained mechanistically.

Gene expression patterns were assigned to 21 functional categories based on the database of clusters of orthologous groups (COG) (Tatusov *et al*., 2000). In total, 3330 of the 3889 genes (86%) could be grouped by COG. The cellular response at maximum ammonia shortage (P5 samples) is indicated by the COG distribution of transcripts determined using the GAGE gene set (Luo *et al*., 2009) and hypergeometric distribution analysis. For each COG category, the resulting *t*‐values are represented in a spider graph (Fig. 3B). Significant changes are defined with a FDR < 0.05. Highly increased transcripts found at the PFR outlet could be linked to energy production and conversion (C), amino acid (E) and carbohydrate (G) transport and metabolism. Decreased gene expression during PFR passage was observed for the genes assigned to ribosomal structure and biogenesis (J), replication, recombination and repair (L), nucleotide (F) and coenzyme (H) transport and metabolism. Maximum expression changes were found in the ‘120 min’ samples and almost returned to starting values after 28 h. In contrast, genes assigned to post‐translational modification, protein turnover and chaperones (O) and cell motility (N) showed increased up‐ and downregulation at 28 h.

Furthermore, we investigated the influence of alternative sigma factors on the observed transcriptional response. In this case, 3461 of 3889 genes (89%) were annotated using the information about sigma factor–gene interactions derived from Regulon DB (Salgado *et al*., 2013). Then, GAGE and hypergeometric analyses were performed in an analogous manner as was used for COG functional testing (Fig. 3C). Notably, genes regulated by the nitrogen stress response sigma factor σ^N^ (RpoN) were typically more abundant at the PFR outlet (FDR < 0.05). Additionally, we found an induction of the σ^S^ (RpoS) regulon, which mediates the general stress response, and an induction of genes that are sensitive for either σ^S^, or the housekeeping factor σ^D^ (RpoD) or ppGpp (Fig. S2A). This was accompanied by the downregulation of transcripts regulated by sigma factor σ^F^ (FliA) which is specific for the transcription of genes involved in cell motility and flagellar synthesis.

### Repeated ammonia shortage triggers transcriptional long‐term responses

**Figure 4:**
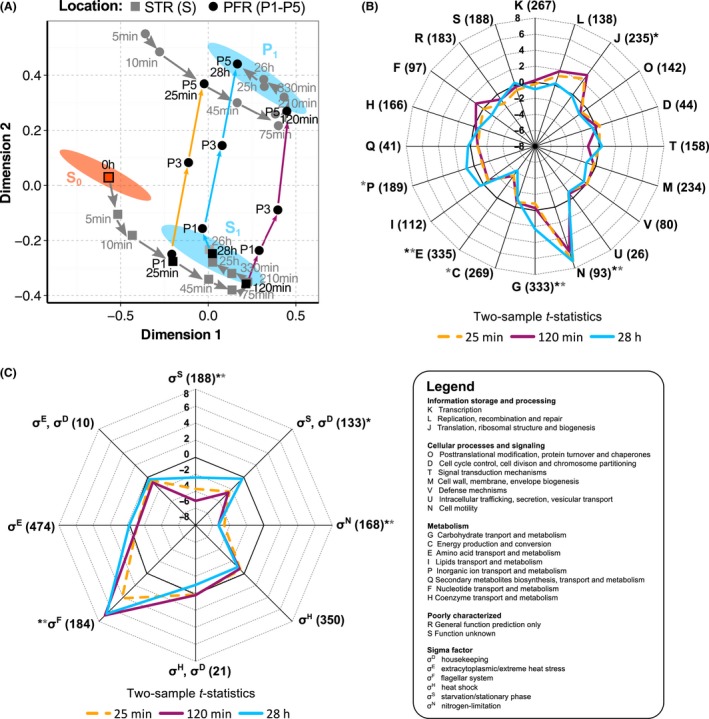
Long‐term dynamics to repeated ammonia shortage. A. Multidimensional scaling analysis of transcriptomes obtained at eleven process time points and over residence time τ in the PFR; Grey arrows follow the adaptation trajectories from the STR (squares) S_0_ (0 h) to S_1_ (28 h) and PFR P5 (circles) and P_1_ (28 h) respectively. Coloured arrows follow the transition between STR and PFR clusters at 25 min (gold), 120 min (magenta) and 28 h (blue). Ellipses indicate the 95% confidence interval of replicate samples taken at S_0_ (orange) and S_1_ at 25, 26 and 28 h after PFR addition (blue). The proportion of variance in the data accounted for by the MDS solution from two independent cultivations: *R* ^2^ = 0.75. Data points represent the mean of *n *=* *2. B. COG functional categories (Tatusov *et al*., 2000) and (C) sigma factor regulation (Salgado *et al*., 2013) pattern for the comparison of STR sample ports S vs. S_0_, visualized as spider graphs. Because no COG or sigma factor annotation was found for 559 and 428 of 3889 genes, respectively, these genes were excluded from the statistical analysis. The *t*‐statistics pattern from GAGE (Luo *et al*., 2009) is shown for three representative time points: 25 min (gold dotted line), 120 min (magenta line) and 28 h (blue line) after PFR addition. Sets including less than 10, or above 500, genes were omitted from the analysis. Functional groups that were significantly changed with an FDR < 0.05 at a minimum of one time point using GAGE (black asterisk) and hypergeometric distribution (grey asterisk) analysis are indicated. Overlapping sigma factor sensitivities: gene regulation may occur by each of the sigma factors depicted because multiple promoters exert control.

After PFR onset (0 h), cells were repeatedly exposed to ammonia shortage in the PFR. Subsequently, they returned to the STR and intermixed with the remaining culture. The adaptation process from the initial steady state S_0_ (STR) to the novel steady state S_1_ in the STR‐PFR was monitored for 28 h by STR sampling and measurement of transcriptional changes as shown in Fig. 4A.

After PFR connection, biomass concentrations and specific uptake rates of ammonia and glucose remained almost constant, but respiratory activity (oxygen uptake and carbon dioxide production rate) increased about 10% (Table S1). Intracellular ppGpp levels fluctuated from around 0.5 μmol g (DW)^−1^ to 1.5 μmol g (DW)^−1^ in the STR (Fig. S1) showing no distinct long‐term trend. In contrast, short‐term accumulation profiles in the PFR were much more pronounced (Fig. 2).

Distinct changes in STR transcriptome patterns were already observed 5 min after PFR connection as is shown in the multidimensional scaling plot (Fig. 4A). The steady state S_0_ before PFR connection was characterized by a 95% confidence ellipse computed from the independent measurements. Transcript dynamics slowed down and finally converged to a new steady state (S_1_) which is defined by a second 95% confidence ellipse based on the samples taken at 25, 26 and 28 h. Notably, there was no overlap between S_0_ and S_1_ confidence ellipses underscoring the existence of transcriptional differences. After 28 h, we identified 60 up‐ and 63 downregulated DEGs compared to S_0_. During the transition from S_0_ to S_1_, 487 genes were found to be differentially expressed to at least one time point with log_2_ fold changes lying between −7.3 and 4.6 (24‐fold up‐ and 153‐fold downregulated). Figure 4A also demonstrates the formation of a new steady state at PFR P5 (P_1_). Thus, transcript dynamics at P5 and in STR follow the same tendency, but with a clear offset. As shown in Fig. 4A, each STR condition can be linked to P5 by tracking the transcript changes along the PFR.

GAGE analysis on COG functional genes sets was performed on STR data. The resulting COG distribution is again depicted for the process times of 25 min, 120 min and 28 h in Fig. 4B. Interestingly, gene expression for amino acid transport and metabolism (E) in S_1_ was lower than in S_0_, which had already occurred 25 min after connecting the PFR. In contrast, cell motility (N) was already markedly upregulated after 10 min and lasted until 28 h. The latter is further confirmed by the increased expression of genes regulated by flagellar sigma factor σ^F^ (Fig. 4C). Transient upregulation was observed for translation, ribosomal structure and biogenesis (J) and carbon transport and metabolism (G). Consistent with the downregulation of amino acid transport and metabolism genes was the reduced long‐term expression of the σ^N^ regulon coordinating the response to nitrogen stress. Moreover, genes of the σ^S^‐mediated stress response were downregulated, which was also true for ppGpp‐controlled genes (Fig. S2B).

### Comparing transcriptional responses on fluctuating ammonia and glucose supply

The short‐ and long‐term responses to fluctuations in ammonia levels were compared to an analogous study examining the effects of fluctuations in glucose levels (Löffler *et al*., 2016) that used an identical experimental design.

**Figure 5:**
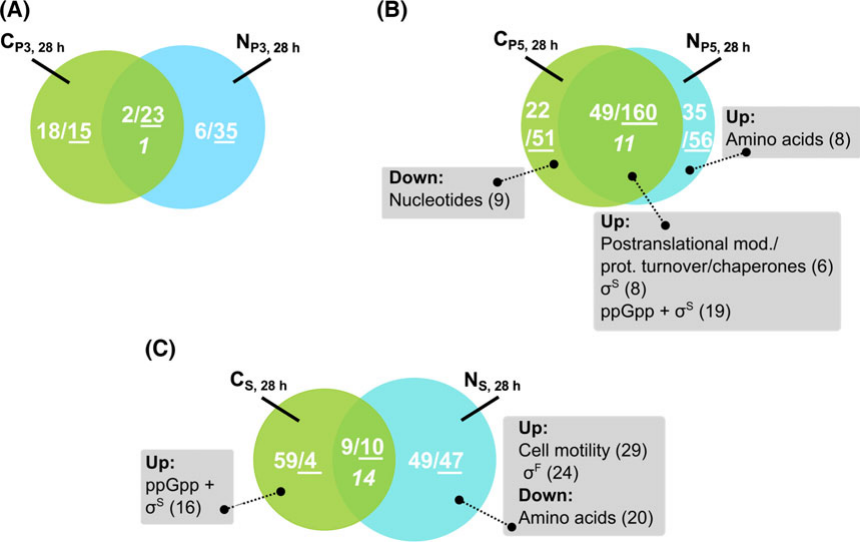
Venn diagrams representing (overlapping) sets of differentially expressed genes derived from repeated ammonia (this study) or glucose shortage (Löffler *et al*., 2016) STR‐PFR tests. Short‐term response observed for the following comparisons at PFR sample port (A) P3 vs. S (τ = 70 s) and (B) P5 vs. S (τ = 110 s) conducted after 28 h of process time. C. Long‐term response (S vs. S_0_) conducted after 28 h. The number of up‐ and downregulated genes in each set is indicated by regular and underlined numbers respectively. The number of overlapping genes, which were regulated in the ammonia and glucose sets with comparable strength but in opposing directions, is shown in italics. For each gene set, a hypergeometric distribution analysis was performed to identify significantly over‐represented functional categories (FDR < 0.05, grey boxes). DEGs were defined as having an FDR < 0.01 and log_2_ fold change ≥ |0.58| in either ammonia or glucose data sets. Complete gene lists of the Venn diagrams are available in Tables S6 ‐ S10 of the Supplementary information.

Strikingly, rapid accumulation of ppGpp in the PFR was detected in both studies even demonstrating similar profiles (Fig. S3). The influence of short‐term starvation stress on gene expression was investigated by comparing the transcriptional profiles of cells sampled after 28 h at PFR sample port P3 or P5 to STR sample port S under glucose and ammonia shortage respectively (for details, see Methods S1). In summary, the expression levels of 25 (25%) and 209 genes (54%) were collectively changed at P3 and P5, respectively, being mostly downregulated (Fig. 5A and B). Consequently, they mirror the transcriptional response to short‐term starvation irrespective of glucose or ammonia shortage. Interestingly, no distinct COG group was found to be dominant. Instead, the 160 genes (Fig. 5B) are distributed over a wide variety of COGs, including biosynthetic (H, F), information processing (J, L, K) and signalling (M, T) pathways. However, common upregulation was found for some genes that are assigned to the ppGpp/σ^S^‐regulon and post‐translational modification, protein turnover and chaperones (Fig. 5B, Figs S4A–S6A). Individual, nutrient‐specific short‐term regulation was observed for 73 ammonia‐ and 91 glucose‐specific genes after 110 s respectively. Additionally, 11 genes were significantly changed under glucose and ammonia limitation but in opposite directions. Among these, amino acid transport and biosynthesis were significantly upregulated under ammonia shortage, whereas nucleotide transport and metabolism were downregulated under glucose shortage.

By analogy, the long‐term transcriptional responses to glucose and ammonia fluctuations were analysed comparing S_28 h_ with S_0_ (Fig. 5C, Methods S1). Only 19 genes (~10%) showed similar long‐term responses under both nutrient conditions, whereas 14 genes were even oppositely regulated under either ammonia or glucose fluctuation. The majority of genes showed nutrient‐specific regulation with 96 (50%) and 63 (33%) genes being primarily differentially expressed under glucose or ammonia respectively. Accordingly, cells cultured under long‐term ammonia fluctuations upregulated cell motility/σ^F^‐dependent genes and downregulated genes associated with amino acid transport and metabolism (Fig. 5C, Figs S4B–6B). In contrast, repeated glucose starvation caused a significant induction of ppGpp/σ^S^‐controlled transcripts. No significant functional groups were found in commonly regulated genes, suggesting that there exists a highly divergent long‐term transcriptional strategy in response to the two nutrient conditions.

## Discussion

### Cellular responses to ammonia shortage

When *E. coli* cells were shifted from ammonia‐limited growth in the STR to full ammonia depletion in the PFR, they repeatedly showed similar short‐term responses irrespective of the process time. The alarmone ppGpp rapidly accumulated in the cells during passage through the PFR, reaching final intracellular concentrations of about 2 μmol g (DW)^−1^ between 70 and 110 s (Fig. 2). Comparable ppGpp responses in response to nitrogen starvation have been reported in other studies (Irr, 1972; Villadsen and Michelsen, 1977; Traxler *et al*., 2011). Nitrogen stress leads to amino acid starvation, which then leads to ribosome stalling because of the presence of uncharged tRNAs, all of which ultimately hampers translation. In turn, the ppGpp synthetase RelA is stimulated to synthesize ppGpp. As the effector molecule of the stringent response, ppGpp allows cells to quickly reprogram transcription in response to nutrient variations (Potrykus and Cashel, 2008). Increased ppGpp levels were already observed at P1 (30 s), suggesting a gradual tactical response to the suboptimal growth conditions as has been proposed by Traxler *et al*. (2011) rather than a classical on/off switch.

The biggest increases in short‐term gene expression were found after 70 s (Fig. 3A), which matched with increasing ppGpp amounts. Nearly all of the DEGs (> 97%) identified at P3 showed ongoing expression until they reached P5 after 110 s. For comparison, no significant up‐ or downregulation of transcripts was observed during the first 30 s. This finding suggests the existence of a cellular ‘reaction time’ of 30–70 s before considerable transcriptional responses are initiated. This conclusion is in line with the similar glucose study (Löffler *et al*., 2016) which also revealed a concerted transcriptional response occurring no earlier than 70 s after glucose shortage. Moreover, the reported timescales are in accordance with those observed in other scale‐down studies (Schweder *et al*., 1999; Lara *et al*., 2006; Buchholz *et al*., 2014).

Analysis of gene expression patterns (Fig. 3C) revealed significant changes in four sigma regulons (σ^S^, σ^N^, σ^F^ and σ^E^) and one overlapping sigma regulon (σ^S^,^D^). A dominant role was found for the σ^S^‐regulon (Gentry *et al*., 1993; Traxler *et al*., 2011) closely networked with ppGpp‐mediated control (Fig. 3B and C, Fig. S2A). The rapid transcriptional upregulation of amino acid transport and metabolism, post‐translational modification, protein turnover and chaperones (E, O in Fig. 3B) as well as catabolic pathways (C, G in Fig. 3B) was accompanied by downregulation of several cellular processes including translation, replication, cell motility and nucleotide/coenzyme biosynthesis (J, L, F, H, N in Fig. 3B). This transcriptional switch is a primary characteristic of the stringent response (Durfee *et al*., 2008; Traxler *et al*., 2008), whereby downregulation of macromolecular synthesis together with altered expression of catabolic genes provides metabolic capacity for amino acid biosynthetic pathways and stress‐protective functions. Nitrogen shortage is known to be primarily sensed as glutamine limitation (Zimmer *et al*., 2000; Gyaneshwar *et al*., 2005b). Accordingly, the limited supply of glutamine required for tryptophan and histidine synthesis seems to be compensated for by the upregulation of corresponding genes such as *trpLE, mtr* and *hisLG*.

Furthermore, σ^N^‐controlled gene expression was found to be amplified with increasing ammonia starvation (Fig. 3C). Many σ^N^‐induced genes are involved in nitrogen assimilation and are additionally controlled by NtrC, the major regulator of the nitrogen‐regulated (Ntr) response (Reitzer and Schneider, 2001; Van Heeswijk *et al*., 2013). Eleven of the 21 known NtrC‐regulated operons (Brown *et al*., 2014) were induced after 110 s at PFR P5 (i.e. *glnK‐amtB, nac, gln and ddp* operons*;* Table S2). Additionally, *zraSR*,* zraP*,* rtcBA*,* rpoH* and the 5′ end genes of the *prp, hyc* and *hyp* operons (Table S3) were also upregulated. These genes seem to be partly involved in nitrogen assimilation under stress conditions (e.g. pH or proton gradient decoupling) (Reitzer and Schneider, 2001). However, other physiological roles more directly connected to nitrogen metabolism have not yet been identified.

In summary, the short‐term response to ammonia limitation started after 30–70 s and consisted mainly of two activities: the initiation of the stringent response (mediated by the concerted control of σ^S^ and ppGpp) and the induction of σ^N^‐dependent Ntr response which is generally described as scavenging response (Zimmer *et al*., 2000). A relationship between both regulatory responses was first discovered by Brown *et al*. (2014) who found that NtrC induces *relA* expression, thereby mediating ppGpp accumulation. Our studies, however, did not reveal increased *relA* levels in the PFR, although intracellular ppGpp levels more than doubled. This observation suggests there are alternative routes (e.g. via uncharged tRNA) for quickly controlling ppGpp levels besides the strategic regulation discovered by Brown *et al*. (2014). In addition, the cellular nitrogen status is sensed by the complex ammonia assimilation system which assesses glutamine and α‐ketoglutarate availabilities, finally leading to the activation of NtrC (Van Heeswijk *et al*., 2013; Chubukov *et al*., 2014). The simultaneous downregulation of transcripts controlled by σ^F^ and σ^E^ may as well be attributed to a fast and complex redistribution of transcriptional capacity (i.e. availability of RNA polymerase) in the favour of σ^S^ and σ^N^‐dependent genes better counteracting ammonia shortage along the PFR.

The basic characteristic of our experimental set up is the frequent recycling of cells from PFR to STR. As a consequence, the regulatory programmes for Ntr stress and the stringent response were only induced briefly and may not even have been completed, because cells were relieved from severe ammonia shortage after 125 s at which time they re‐entered the STR. Within this time, the ppGpp response was induced, but may not have yet reached maximum levels which would take between 10 to 20 min as shown by Wang *et al*. (2007) and Traxler *et al*. (2011). Yet, the relatively small ppGpp increase already induced transcriptional changes immediately. Similar studies with glucose (Löffler *et al*., 2016) revealed a frequent on/off switching of several hundred genes between the bioreactor compartments. Our study unravelled the repeated up‐ and downregulation of about 250 and 100 genes respectively. Although glucose fluctuations caused an increase in ATP maintenance demands of 40–50%, nitrogen stress increased ATP demands by only 15% (for details see Methods S2). This finding underscores the fact that glucose limitation affects both catabolic and anabolic functions, whereas ammonia limitation mainly affects solely anabolism. Of particular note, genes with high on/off switching costs were mainly associated with amino acid transport and metabolism (E) and nitrogen regulation (e.g. *glnK, glnH, trpLE, hisG* in Table S4) underscoring the fact that cells have to expend energetic effort to compensate for ammonia shortage.

Transcript analysis of samples at P5 showed that only the genes closest to the operon 5′ end were significantly upregulated in large operons. This likely illustrates the time restrictions for transcription changes to occur in the PFR. However, evidence was found that transcription, once initiated in the PFR, can propagate into the STR. Examples are the *trp* and *his* operons which were strongly induced in the PFR. In both cases, increases in the gene expression levels of the genes downstream of *trpLE* and *hisLG* were found in the STR, which highlights that transcription, must have continued in the STR after it had been initiated in the PFR. Further studies to model this phenomenon are ongoing.

### Long‐term adaption to repeated ammonia shortage

Over the long‐term, repeated periods of ammonia shortage caused a switch from the transcriptional steady state S_0_ to S_1_ in the STR (Fig. 4A). A detailed analysis of STR gene expression patterns revealed that S_1_ mainly differs from S_0_ by σ^F^‐dependent induction of genes involved in cell motility (N), carbohydrate metabolism (G) and repression of amino acid transport and metabolism (E), as well as repression of σ^N^ and σ^S^‐induced genes (blue lines, Fig. 4B and C). During the transition from S_0_ to S_1_, we found transient upregulation of translation and ribosome biogenesis (J). Apparently, transcriptional programmes that were initiated in the PFR were repeatedly counter‐acted in the STR. ATP demands required for these changes are relatively low, so that *E. coli* can energetically afford frequent reprogramming of nitrogen responses.

In principle, *E. coli* is able to quickly reset regulatory responses. For example, Gyaneshwar *et al*. (2005a) found decreased transcription of genes involved in cell motility during downshifts in nitrogen or sulphur levels and subsequent upregulation during nitrogen or sulphur upshifts. This behaviour resembles our short‐ and long‐term observations under ammonia fluctuations. As a first line of defence against ammonia starvation (short‐term response), *E. coli* uses its capacities to utilize nitrogen‐containing compounds (Zimmer *et al*., 2000) rather than to seek new sources (Ntr stress response). Moreover, σ^D^‐dependent transcription of the *flhDC* operon that encodes the master regulator of the flagellar cascade is directly inhibited by ppGpp (Lemke *et al*., 2009), which is consistent with our PFR data showing on average 1.5‐ to twofold downregulation of *flhDC*. This mechanism may be essential under very poor nutritional conditions and also in the stationary phase. However, under the frequently changing conditions of large bioreactors, mirrored in our experimental set‐up, it causes the frequent re‐induction of flagella and chemotaxis genes under the less stressful STR conditions (long‐term response). In particular, genes for chemotaxis receptors mediating taxis towards amino acids and dipeptides (*tsr, tap* and *tar*) were upregulated (Fig. S7).

Hierarchical expression of flagella and chemotaxis genes commenced after 5 min with the induction of the master operon encoding FlhDC*,* which then initiated the downstream cascade (Fig. S7). As late filament and taxis genes were significantly upregulated 45 min after PFR addition, we conclude that the essential hook basal body, which forms a developmental checkpoint for their induction (Chilcott and Hughes, 2000), is functionally present. Accordingly, cellular maintenance demands were likely to increase because of the additional efforts required for flagella synthesis and cell motility (Macnab, 1996). While wild‐type strains may be well prepared to compensate for their additional ATP needs, high‐producer strains with additional, product‐driven demands for ATP and other building blocks may reach energetic limits (Löffler *et al*., 2016).

### Characteristics of periodically changing ammonia or glucose availability

The implementation of glucose or ammonia shortage in the PFR caused ppGpp responses characterized by a gradual accumulation beginning after 30 s and subsequent transcriptional changes being observed after about 70 s. Even though the relative increase in ppGpp level (~threefold) and profiles were alike under both, ammonia and glucose nutrient conditions, ppGpp basal levels measured in the STR reached higher values under ammonia fluctuations (Fig. S3). Under intermediate nutrient limitation, slightly elevated ppGpp levels are known to be maintained (Traxler *et al*., 2011); differences may then be related to the type of substrate stress reflecting the homoeostatic balance under the respective nutrient conditions.

Short‐term ammonia and glucose transcriptional responses resembled each other with increasing residence time in the PFR (Fig. S8, Fig. 5A and B). After 110 s, 54% of the genes showed comparable transcriptional responses (Fig. 5B). Within this group, ppGpp‐mediated regulation turned out to be a common strategy, which is exemplified by significant induction of several ppGpp/σ^S^‐dependent genes. Accordingly, the stringent response encompassed downregulation of several genes related to translation, replication, cell wall/membrane biogenesis and signal transduction as well as nucleotide/coenzyme transport and metabolism. This indicates the rapid induction of a general protective starvation response that is independent of the original limitation (hunger response) as proposed by Ferenci (2001).

We also identified nutrient‐specific regulation in the PFR (46% of the genes in Fig. 5B), i.e. enhanced upregulation of amino acid biosynthesis and transport and downregulation of nucleotide biosynthesis and transport. Such nutrient‐specific regulatory programmes mirror the individual interaction between nitrogen limitation and anabolic functions (Zimmer *et al*., 2000) and carbon limitation with catabolism and energy (Löffler *et al*., 2016).

Unlike short‐term regulation, the long‐term transcriptional responses revealed fundamental differences in cellular strategies to adapt to fluctuating ammonia and glucose supply. Only 19 genes (10%) showed similar expression ratios under both conditions (Fig. S8, Fig. 5C). Although the ppGpp‐mediated stringent response was induced in the PFR for both glucose and ammonia triggers, only in the glucose study did a likewise long‐term regulatory programme became manifest (Löffler *et al*., 2016) (Fig. 5C). In the case of nitrogen, short‐term induction of the stringent response stimuli was reset for long‐term transcriptional adaptation (Figs 2A, 3C and 4C). Although the general stress response plays an essential part during ammonia depletion (Gyaneshwar *et al*., 2005a), σ^S^ levels under ammonia shortage were found to be only slightly increased and were lower than those seen in glucose starvation (Mandel and Silhavy, 2005). Unlike upon glucose starvation, σ^S^ is not stabilized upon ammonia starvation suggesting that cells can modulate σ^S^ activity under nitrogen shortage (Mandel and Silhavy, 2005; Peterson *et al*., 2005). Accordingly, following periodic glucose shortage, stabilized σ^S^ may accumulate over time thereby mediating propagation of the signal into the STR. In contrast, the quick modulation of σ^S^ activity seen under ammonia deprivation may open the door to other adaptation programmes. In this context, the broad induction of cell motility/σ^F^ genes, which was the most prominent long‐term response specific to repeated ammonia starvation (Figs  4B–C and 5C), may present a strategic cellular reaction to actively seek for nitrogen sources in response to environmental ammonia gradients. Additionally, it may be interesting whether genetic mutations may have occurred during the course of the experiments. Therefore, variant calling was performed based on the RNA‐seq data. However, only one non‐synonymous variant was identified in the coding region for an uncharacterized protein YccE after 28 h of cultivation in the STR‐PFR system (Methods S4, Table S5).

In summary, ammonia and glucose gradients in the PFR induce strong transcriptional responses after 70 s, which remarkably resemble those seen after 110 s. However, the long‐term strategies for adaptation differ significantly between ammonia and glucose starvation. This may be caused by differences in σ^S^ stabilities under the two conditions. The cellular needs required to adapt to ammonia fluctuations are less costly than for glucose, which may encourage the application of related scenarios for the control of production processes on a large scale.

## Experimental procedures

### Bacterial strain, pre‐culture and media

In this study, bacterial cultivations were performed using the *Escherichia coli* K‐12 W3110 LJ110 strain (Zeppenfeld *et al*., 2000; Trachtmann *et al*., 2016), kindly provided by G. Sprenger (University of Stuttgart). Baffled shaking flasks (2 l) containing 300 ml of minimal media were inoculated with glycerol stock seed cultures. The minimal media consisted of (per litre) 4 g glucose, 3.2 g NaH_2_PO_4._· 2 H_2_O, 11.7 g K_2_HPO_4_, 8 g (NH_4_)_2_SO_4_, 0.01 g thiamine, and a trace element solution (0.11 g Na_3_C_6_H_5_O_7_, 0.00835 g FeCl_3_· 6 H_2_O, 0.00009 g ZnSO_4_· 7 H_2_O, 0.00005 g MnSO_4_· H_2_O, 0.0008 g CuSO_4_· 5 H_2_O, 0.00009 g CoCl_2_· 6 H_2_O, 0.0044 g CaCl_2_· 2 H_2_O, 0.1 g MgSO_4_· 7 H_2_O). Pre‐cultures were grown overnight at 37°C with agitation (130 rpm).

### Batch and STR‐PFR chemostat cultivations

One hundred and fifty millilitres of pre‐culture were used as an inoculum for the batch bioreactor cultivation. The bioreactor cultivations were performed using a two‐compartment bioreactor system consisting of an STR with a recycle loop (PFR) as described in Löffler *et al*. (2016). The STR volume was 3 l (Bioengineering, Wald, Switzerland) with a 1.5 l working volume, a constant aeration rate (1.5 l min^−1^) and a total pressure of 1.5 bar. The reactor was equipped with a six‐blade Rushton type impeller applying a constant power input of 5 W. pH and pO_2_ were measured using Mettler Toledo (Columbus, OH, USA) and PreSens (Regensburg, Germany) probes respectively. A pH set point of 7.0 was adjusted using 3 M NaOH and 2.5 M H_3_PO_4_ and broth temperature was maintained at 37°C. To preserve foam formation, 50 μL h^−1^ antifoam (Struktol J 647; Schill+Seilacher, Hamburg, Germany) was added constantly during the chemostat phase. Bioreactor cultivations were carried out with a minimal medium containing (per litre) 19.0 g glucose, 1.0 g NaH_2_PO_4_.· 2 H_2_O, 2.6 g K_2_HPO_4_, 3.8 g (NH_4_)_2_SO_4_ and a trace element solution with the same composition as that used in the shaking flask minimal medium. A constant dilution rate (*D* = 0.2 h^−1^) was established after the end of the batch phase and was verified via dissolved oxygen and off‐gas analysis levels. Circulation through the PFR was started, after steady‐state conditions were achieved in the STR (reached after five residence times). The continuous recirculation flow of the bio‐suspension from the STR to the PFR was provided using a diaphragm metering pump (Sigma/1 S1Cb, ProMinent, Heidelberg, Germany) and flow was measured using a Coriolis flow meter (Cubemass DCI RS‐485, Endress+Hauser, Weil am Rhein, Germany).

The combined working volume was maintained at a constant 1.5 l. The volumetric proportion was 75% in the STR (*V*_STR_ = 1.12 l) and 25% in the PFR (*V*_PFR_ = 0.38 l). The PFR, with an inner tube diameter of 20 mm, features five sample ports with an additional air sparger placed at sample port P1 to ensure an oxygen saturation over 10% (air flow: 0.15 l min^−1^). Dissolved oxygen was controlled close to sample ports P1 and P5. The broth temperature in the PFR was kept constant at 37°C using a curing bag (Calorex EPDM, Chemietechnik GmbH and Co., Heidelberg, Germany) and isolation material (HT Armaflex, Armacell International S.A, Luxembourg, Luxembourg). Process control and data processing were performed using LabVIEW^®^ 2010 (National Instruments, Austin, TX, USA). All samples were taken from the STR‐PFR system using a low‐dead‐volume rapid sampling device consisting of a steam sterilizable miniature valve coupled to a HPLC capillary (Theobald *et al*., 1993). Additionally, overpressure in the system ensured rapid transfer of the bio‐suspension into the sampling tube. Cells passed the device in < 0.75 s and were harvested into pre‐cooled sampling tubes containing the stabilizing reagents.

### Biomass, ammonia and phosphate determination

Biomass concentration was quantified gravimetrically in quadruplicate as described in Löffler *et al*. (2016). The extracellular substrates ammonia and phosphate were measured using Hach Lange Kits LCK 348 and LCK 303 (Hach Lange, Duesseldorf, Germany) respectively, according to the manufacturer's protocol.

### Organic acid and ppGpp quantification

An isocratic HPLC equipped with an RI detector (1200 Series; Agilent, Santa Clara, CA, USA) and a Rezex ROA‐Organic Acid H^+^ (300 × 7.8 mm, 8 μm, Phenomenex, Torrance, CA, USA) column, protected by a Rezex ROA guard column Carbo‐H (50 × 7.8 mm, Phenomenex), was used for the quantification of glucose and the by‐products acetic acid, succinic acid, lactic acid, formic acid and ethanol. Sample preparation was performed as described by Buchholz *et al*. (2014).

ppGpp was extracted from 2 ml of bio‐suspension by direct sampling into 0.5 ml precooled (−30°C) perchloric acid (35% (v/v)) and incubated with shaking for 15 min at 6°C (Theobald *et al*., 1997; Cserjan‐Puschmann *et al*., 1999). The pH of the samples was neutralized by adding KH_2_PO_4_ and KOH. After centrifugation (15 min, 4°C, 7000 *g*), the supernatant was collected and analysed via HPLC (1200 Series; Agilent, Santa Clara, CA, USA) equipped with a RP‐C18 (octadecyl) phase column (Supercosil™ LC‐18‐T, 3 μm, 150 × 4.6 mm) and a diode array detector (DAD). Gradient elution was performed at a flow rate of 1 ml min^−1^ using buffer A (0.1 M KH_2_PO_4_, 0.1 M K_2_HPO_4_, 4 mM TBAS, pH 6) and buffer B (0.1 M KH_2_PO_4_, 0.1 M K_2_HPO_4_, 4 mM TBAS, pH 7.2 + 30% methanol) to produce the following gradient: 3.5 min, 0% B; 20 min, 30% B; 22 min, 35% B; 40 min, 60% B; 48 min, 100% B; 55 min, 100% B; 60 min 0% B; 67 min, 0% B. Quantification of ppGpp was conducted using a 7‐point external calibration curve with a ppGpp standard (TriLink BioTechnologies, San Diego, CA, USA). In all analytical steps, special attention was given to the continuous cooling of samples (< 6°C).

### RNA‐sequencing analysis

Cell samples taken from independent duplicate biological STR‐PFR cultivations were used for gene expression analysis. Sampling was performed by placing the culture sample directly into RNAprotect Bacteria Reagent (Qiagen, Hilden, Germany) and the pellet was stored at −70°C after centrifugation. The RNeasy mini kit, including on column RNase‐Free DNase I treatment (both Qiagen), was used for total RNA isolation (RNA ≥ 200 bases), according to the manufacturer's protocol. RNA quality was determined by a Lab‐on‐a‐Chip‐System Bioanalyzer 2100 (Agilent, Boeblingen, Germany). For library preparation, ribosomal RNA was depleted from 1 μg of total RNA using the Ribo‐Zero™ Magnetic Kit (Bacteria) (Epicentre, Madison, WI, USA). Next, mRNA libraries were prepared using the TruSeq mRNA Library Prep Kit (Illumina, San Diego, CA, USA) according to the manufacturer's instructions. Library size (approximately 400 bp) was confirmed using the Bioanalyzer 2100 and the concentration (approximately 40 ng/μl) was determined using Qubit Fluorometric Quantitation (Thermo Fisher Scientific, Waltham, MA, USA). The library was denaturated according to the manufacturer's instructions and diluted to 9 pM. Samples were sequenced on an Illumina HiSeq 2500 platform (Illumina, San Diego, CA, USA) in the HighOutput mode (68 cycles, single end reads), sequencing approximately 10 million clusters per sample. The blc2fastq Conversion Software v. 1.8.4 from Illumina (http://support.illumina.com/downloads.html) was used to translate cluster intensity values into fastq files. Cutadapt v. 1.8.3 (Martin, 2011) was used to remove remaining sequencing adapter bases for the following alignment step. As a reference, reads were aligned against the NCBI *E. coli* K12 W3110 genome (GenBank: AP009048.1) using the RNA‐sequencing aligner STAR v. 2.4.2a (Dobin *et al*., 2013), resulting in 98% mapped reads (on average). Aligned reads were counted for each gene based on the respective annotations available from UCSC genome browser (http://genome.ucsc.edu) for the chosen reference sequence applying HTseq‐count v. 0.6.1 (Anders *et al*., 2015) in the intersection‐nonempty mode. On average 94% of the sequenced reads could be assigned uniquely to annotated features making up about 9 ± 1.4 million reads per sample covering approximately 90% of all annotated genes by at least 10 reads.

### Transcriptome data analysis

Differential gene expression analysis was performed with the r‐package edgeR v. 3.8.6 (Robinson *et al*., 2010), downloaded from Bioconductor (Gentleman *et al*., 2004; http://www.bioconductor.org). HTseq‐derived raw counts were used as input, after removal of residual rRNA and tRNA molecules and a non‐specific filtering step to remove low coverage genes with fewer than two counts per million (16–20 reads) in more than 25% of the dataset. The design matrix was set up by grouping the samples by replicates and combining sample time and location (STR or PFR) into one experimental factor. A negative binominal was fitted to the data and the robust genewise dispersions were estimated (Zhou *et al*., 2014). Then, a generalized linear model was fitted to the data using the estimated dispersions and design. Differential expression for a given contrast was tested using genewise likelihood ratio tests. Multiple hypothesis testing correction was performed on the resulting *P*‐values, to control the false discovery rate (FDR) according to Benjamini and Hochberg (1995). Genes were regarded as significantly differentially expressed with FDR adjusted *P*‐values < 0.01 and log_2_ fold changes ≥ |0.58|. The reproducibility of biological replicates was estimated by calculating Spearman's rank correlation coefficient. In this way, three outlier observations (PFR P5 75 min/330 min/26 h) were removed from the data set. The outliers contained extremely high counts of some transcripts which can be an issue in RNA samples within an experimental group (Zhou *et al*., 2014; George *et al*., 2015). As a measure of mRNA abundance in a sample, the estimated fraction of transcripts formed by a distinct gene was computed in R and scaled by multiplying by 10^6^ to get transcripts per million (TPM) as previously described (Li and Dewey, 2011).

Based on their TPM values, the 600 genes with the largest standard deviation between samples were selected for multidimensional scaling (MDS) analysis. Metric MDS was performed using edgeR's plotMDS function (Robinson *et al*., 2010) with Euclidean distance as a proximity measure. Confidence ellipses were drawn by estimating the covariance matrix, assuming the data came from a multivariate *t*‐distribution with a confidence level of 95% (Fox and Weisberg, 2010). The coefficient of determination *R* ^2^ was obtained by computing and squaring the correlation between the original distances and the distances determined from the two‐dimensional MDS solution_._

Gene set enrichment and over‐representation analysis of clusters of up‐ and downregulated genes were performed using Bioconductor's R‐package GAGE v. 2.16.0 (Luo *et al*., 2009) and R's Hypergeometric test respectively. With both methods, gene sets were selected as significantly different with an FDR adjusted *P*‐value < 0.05 (Benjamini and Hochberg, 1995). Functional annotation for the statistical tests was derived from the Cluster of Orthologous Groups (COG) database (Tatusov *et al*., 2000; http://www.ncbi.nlm.nih.gov/COG, last modified: 4‐2‐2015), the experimental sigma factor–gene interaction dataset from RegulonDB v. 8.0 (Salgado *et al*., 2013) (http://regulondb.ccg.unam.mx/, last modified: 9‐15‐2015) as well as a list of genes requiring ppGpp, σ^S^ and Lrp for their induction identified by mutant studies (Traxler *et al*., 2011). The RNA‐sequencing data derived from periodic ammonia stimulation experiments have been deposited in NCBI's Gene Expression Omnibus (GEO) (Edgar *et al*., 2002) and are accessible through GEO series accession number GSE90743 (https://www.ncbi.nlm.nih.gov/geo/query/acc.cgi?acc=GSE90743). Raw count and processed data from the glucose tests can be found in the Supplementary information of Löffler *et al*. (2016). Data analysis was performed using the free statistical computing environment r v. 3.1.3.

## Conflict of interest

None declared.

## Supporting information


**Fig. S1.** Time profiles of the intracellular ppGpp concentration over process time sampled at STR (squares) and PFR P5 (circles) from two independent biological cultivations.
**Fig. S2.** Box plots of log_2_ fold changes for each group of genes induced by ppGpp (Traxler *et al*., 2011) at (A) each sample port P1‐P5 along the PFR and (B) over process time in the STR.
**Fig. S3.** Intracellular ppGpp levels measured at each sample port in ammonia (gold) and glucose (grey) STR‐PFR stimulation experiments.
**Fig. S4.** Bar plots of COG distributions (Tatusov *et al*., 2000) for (A) short‐term (P5 vs. S) and (B) long‐term (S vs. S_0_) impacts of genes showing similar (ΔN ≈ ΔC) or nutrient‐dependent (ΔN ≫ ΔC, ΔN ≪ ΔC) transcriptional responses after 28 h under ammonia and glucose shortage, respectively (for details see Supplementary methods S1).
**Fig. S5.** Bar plots of alternative sigma factor interactions (Salgado *et al*., 2013) for (A) short‐term (P5 vs. S) and (B) long‐term (S vs. S_0_) impacts of genes showing similar (ΔN ≈ ΔC) or nutrient‐dependent (ΔN ≫ ΔC, ΔN ≪ ΔC) transcriptional responses after 28 h under ammonia and glucose shortage, respectively (for details see Supplementary methods S1).
**Fig. S6.** Bar plots of ppGpp, σ^S^ and Lrp regulons and interactions from (Traxler *et al*., 2011) for (A) short‐term (P5 vs. S) and (B) long‐term (S vs. S_0_) impacts of genes showing similar (ΔN ≈ ΔC) or nutrient‐dependent (ΔN ≫ ΔC, ΔN ≪ ΔC) transcriptional responses after 28 h under ammonia and glucose shortage, respectively (for details see Supplementary methods S1).
**Fig. S7**. Heat map of log_2_ fold change values for flagellar and chemotaxis genes (*n *=* *52) that significantly changed in expression over process time in the STR.
**Fig. S8.** Scatter plots of short‐ (upper panel) and long‐term (lower panel) ammonia and glucose logarithmic expression ratios.
**Fig. S9**. PFR tracer curves.
**Fig. S10.** (A) Short‐term (P5 vs. S) and (B) long‐term (S vs. S_0_) distributions of absolute differential expression ratios between ammonia and glucose conditions (δ  =  |log_2_ (Δ_N_)| – |log_2_ (Δ_C_)|) after 28 h.
**Fig. S11.** Schematic outline of additional maintenance requirements due to transcriptional changes along the PFR.
**Table S1.** Summary of process parameters including oxygen transfer rate (OTR), carbon transfer rate (CTR), the respiratory quotient (RQ) and specific oxygen uptake (qO_2_), glucose (q_glc_), ammonia (q_am_) uptake and carbon dioxide consumption (qCO_2_) rates.
**Table S2.** Logarithmic expression ratios of σ^N^‐dependent genes/operons involved in the nitrogen regulatory (Ntr) response between sample port PFR P5 and STR S.
**Table S3.** Logarithmic expression ratios of σ^N^ dependent genes/operons not directly involved in nitrogen metabolism between sample port PFR P5 and STR S.
**Table S4.** Differentially expressed genes (FDR <0.01) with the highest estimated energy demands for transcription and translation.
**Table S5**: List of variants identified after 28 h of cultivation in the STR‐PFR system.
**Methods S1.** Plug flow reactor characterization
**Methods S2.** Gene grouping for the comparison of ammonia and glucose transcriptome data
**Methods S3.** Estimation of ATP cost of gene expression
**Methods S4.** Genetic variant discovery

**Table S6.** Venn diagram gene list short‐term response (P3 vs. S) after 28 h
**Table S7.** Venn diagram gene list short‐term response (P5 vs. S) after 28 h
**Table S8.** Venn diagram gene list long‐term response (S vs. S0) after 28 h
**Table S9.** Differentially expressed gene list short‐term response (P5 vs. S) after 28 h
**Table S10.** Differentially expressed gene list long‐term response (S vs. S0) after 28h
