# Supplementary material for: Transcriptional response of Escherichia coli to ammonia and glucose fluctuations
Source: Microb Biotechnol. 2017 Apr 26;10(4):858–72. doi: 10.1111/1751-7915.12713 (PMC5481515; doi:10.1111/1751-7915.12713)
Supplement: Supplementary file 1 — Fig. S1. Time profiles of the intracellular ppGpp concentration over process time sampled at STR (squares) and PFR P5 (circles) from two independent biological cultivations. Fig. S2. Box plots of log2 fold changes for each group of genes induced by ppGpp (Traxler et al., 2011) at (A) each sample port P1‐P5 along the PFR and (B) over process time in the STR. Fig. S3. Intracellular ppGpp levels measured at each sample port in ammonia (gold) and glucose (grey) STR‐PFR stimulation experiments. Fig. S4. Bar plots of COG distributions (Tatusov et al., 2000) for (A) short‐term (P5 vs. S) and (B) long‐term (S vs. S0) impacts of genes showing similar (ΔN ≈ ΔC) or nutrient‐dependent (ΔN ≫ ΔC, ΔN ≪ ΔC) transcriptional responses after 28 h under ammonia and glucose shortage, respectively (for details see Supplementary methods S1). Fig. S5. Bar plots of alternative sigma factor interactions (Salgado et al., 2013) for (A) short‐term (P5 vs. S) and (B) long‐term (S vs. S0) impacts of genes showing similar (ΔN ≈ ΔC) or nutrient‐dependent (ΔN ≫ ΔC, ΔN ≪ ΔC) transcriptional responses after 28 h under ammonia and glucose shortage, respectively (for details see Supplementary methods S1). Fig. S6. Bar plots of ppGpp, σS and Lrp regulons and interactions from (Traxler et al., 2011) for (A) short‐term (P5 vs. S) and (B) long‐term (S vs. S0) impacts of genes showing similar (ΔN ≈ ΔC) or nutrient‐dependent (ΔN ≫ ΔC, ΔN ≪ ΔC) transcriptional responses after 28 h under ammonia and glucose shortage, respectively (for details see Supplementary methods S1). Fig. S7. Heat map of log2 fold change values for flagellar and chemotaxis genes (n = 52) that significantly changed in expression over process time in the STR. Fig. S8. Scatter plots of short‐ (upper panel) and long‐term (lower panel) ammonia and glucose logarithmic expression ratios. Fig. S9. PFR tracer curves. Fig. S10. (A) Short‐term (P5 vs. S) and (B) long‐term (S vs. S0) distributions of absolute differential expression ratios between am [file MBT2-10-858-s001.docx]

# SUPPLEMENTARY MATERIAL:

***Transcriptional Response of Escherichia coli to Ammonia and Glucose Fluctuations***

# Author names and affiliations:

Joana Danica Simen^1^, Michael Löffler^1^, Günter Jäger^2^, Karin Schäferhoff^2^, Andreas Freund^1^, Jakob Matthes^2^, Jan Müller^1^, RecogNice-Team^¶^, Ralf Takors^1,^*

^1^ University of Stuttgart, Institute of Biochemical Engineering, Allmandring 31, 70569 Stuttgart, Germany

^2^ University of Tübingen, Institute of Medical Genetics and Applied Genomics, Calwerstr. 7, 72076 Tübingen, Germany

* For correspondence.

*E-mail address*: takors@ibvt.uni-stuttgart.de;

*Tel.*: (+49) 711 685 64535;

*Fax*: (+49) 711 685 65164

^¶^ For membership list refer to Acknowledgements section.

# Supplementary Figures

|  |
| --- |
| **Fig. S1.** Time profiles of the intracellular ppGpp concentration over process time sampled at STR (squares) and PFR P5 (circles) from two independent biological cultivations. Start of PFR connection is indicated by an arrow. STR data at time 0 h represent mean ± s.d. from three samples collected from S_0_ over 16 hours. |

|  |
| --- |
| **Fig. S2.** Box plots of log_2_ fold changes for each group of genes induced by ppGpp (Traxler *et al.*, 2011) at (A) each sample port P1-P5 along the PFR and (B) over process time in the STR. Distributions which significantly (FDR < 0.05) deviate from zero as tested by GAGE (Luo *et al.*, 2009) are indicated with an asterisk. |

|  |
| --- |
| **Fig. S3.** Intracellular ppGpp levels measured at each sample port in ammonia (gold) and glucose (grey) STR-PFR stimulation experiments. Each bar represents the respective average ppGpp concentration in µmol g (DW)^-1^ for the independent cultivation at process times 25 min, 120 min and 28 h (mean ± s.d). |

|  |
| --- |
| **Fig. S4.** Bar plots of COG distributions (Tatusov *et al.*, 2000) for (A) short-term (P5 vs. S) and (B) long-term (S vs. S_0_) impacts of genes showing similar (ΔN ≈ ΔC) or nutrient-dependent (ΔN >> ΔC, ΔN << ΔC) transcriptional responses after 28 h under ammonia and glucose shortage, respectively (for details see Supplementary methods S1). For each COG the number of differentially expressed genes (DEGs, black) and non-DEGs (NDGs, white) is indicated. COGs significantly enriched (FDR < 0.05) according to hypergeometric distribution analysis are indicated with an asterisk (red). DEGs with FDR < 0.01 and log2 fold change ≥ \|0.58\|. COGs: K, Transcription; L, Replication, recombination and repair; J, Translation, ribosomal structure and biogenesis; O, Posttranslational modification, protein turnover and chaperones; D, Cell cycle control, cell division and chromosome partitioning; T, Signal transduction mechanisms; M, Cell wall, membrane, envelope biogenesis; V, Defense mechanisms; U, Intracellular trafficking, secretion, vesicular transport; N, cell motility; G, Carbohydrate transport and metabolism; C, energy production and conversion; E, Amino acid transport and metabolism; I, Lipids transport and metabolism; P, Inorganic ion transport and metabolism; Q, Secondary metabolites biosynthesis, transport and metabolism; F, Nucleotide transport and metabolism; H, Coenzyme transport and metabolism; R, General function prediction only; S, Function unknown. |

|  |
| --- |
| **Fig. S5.** Bar plots of alternative sigma factor interactions (Salgado *et al.*, 2013) for (A) short-term (P5 vs. S) and (B) long-term (S vs. S_0_) impacts of genes showing similar (ΔN ≈ ΔC) or nutrient-dependent (ΔN >> ΔC, ΔN << ΔC) transcriptional responses after 28 h under ammonia and glucose shortage, respectively (for details see Supplementary methods S1). For each sigma factor interaction the number of differentially expressed genes (DEGs, black) and non-DEGs (NDGs, white) is indicated. Distributions that were significantly enriched in up- or downregulated genes (FDR < 0.05) according to hypergeometric distribution analysis are indicated with an asterisk (red). DEGs with FDR < 0.01 and log2 fold change ≥ \|0.58\|. Sigma factors: σ^Fer^, Ferric citrate, σ^E^, Extracytoplasmic/extreme heat stress; σ^F^, Flagellar system; σ^H^, Heat shock; σ^S^, Starvation/stationary phase; σ^N^, Nitrogen limitation, σ^D^, Housekeeping. |

|  |
| --- |
| **Fig. S6.** Bar plots of ppGpp, σ^S^ and Lrp regulons and interactions from (Traxler *et al.*, 2011) for (A) short-term (P5 vs. S) and (B) long-term (S vs. S_0_) impacts of genes showing similar (ΔN ≈ ΔC) or nutrient-dependent (ΔN >> ΔC, ΔN << ΔC) transcriptional responses after 28 h under ammonia and glucose shortage, respectively (for details see Supplementary methods S1). For each regulon the number of differentially expressed genes (DEGs, black) and non-DEGs (NDGs, white) is indicated. Distributions that were significantly enriched in up- or downregulated genes (FDR < 0.05 according to hypergeometric distribution analysis are indicated with an asterisk (red). DEGs with FDR < 0.01 and log_2_ fold change ≥ \|0.58\|. |

|  |
| --- |
| **Fig. S7**. Heat map of log_2_ fold change values for flagellar and chemotaxis genes (*n* = 52) that significantly changed in expression over process time in the STR. The dendrogram shows the results of hierarchical clustering (average linkage) using Pearson correlation as distance measure between genes. The observed transcriptional hierarchy closely follows the temporal classes defined by Chilcott and Hughes, (2000). Transcription units (TUs) are color coded. DEGs with FDR < 0.01 and log_2_ fold change ≥ \|0.58\| in more than one sample. |

|  |
| --- |
| **Fig. S8.** Scatter plots of short- (upper panel) and long-term (lower panel) ammonia and glucose logarithmic expression ratios. Pearson’s correlation coefficient (r) and trend line (red) represent the strength and direction of the linear relationship between the two global expression profiles. Genes are color coded according to the Venn diagram in Figure 5 and the grouping described in Supplementary methods S1. |

|  |
| --- |
| **Supplementary Fig. S9**. PFR tracer curves. Residence times for each PFR sample port were derived from the tracer curves and the corresponding curves are color coded. |

|  |
| --- |
| **Fig. S10.** (A) Short-term (P5 vs. S) and (B) long-term (S vs. S_0_) distributions of absolute differential expression ratios between ammonia and glucose conditions (δ = \|log_2_ (Δ_N_)\| – \|log_2_ (Δ_C_)\|) after 28 h. The selection limits (-1σ < δ < +1σ) for group A genes are indicated by orange dotted lines. Green dotted lines indicate selection limits for group B genes (δ ≤ -2σ or δ ≥ +2σ). |

| 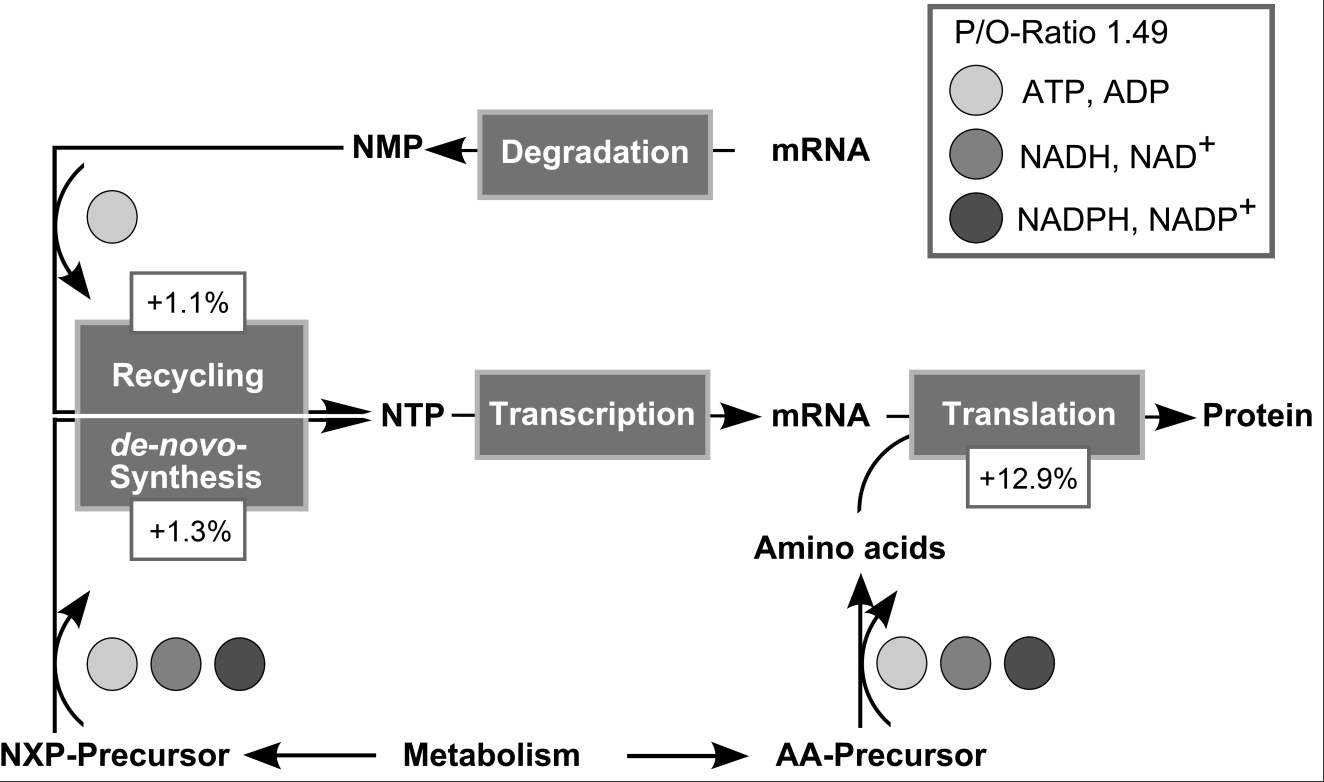 |
| --- |
| **Fig. S11.** Schematic outline of additional maintenance requirements due to transcriptional changes along the PFR. NTP demand or NMP release was calculated for every gene with a significant expression change between the PFR and the STR (FDR < 0.01). If the total NTP demand exceeded the NMP release, de *novo* synthesis costs were used to calculate the energy demand for the newly synthesized nucleotides. For nucleotide recycling, an energy demand of 2 ATP per nucleotide was assumed. The overall costs for translation arise from amino acid synthesis and polymerization, including tRNA loading. It was assumed that each mRNA is translated 11 times. The average costs (gray box) for recycling, *de novo* synthesis, and translation were calculated as a percentage of the growth-decoupled maintenance, assuming a P/O ratio of 1.49 and equivalent energy content for NADH and NADPH. For details on calculations see Supplementary Methods S1. |

# Supplementary Tables

**Table S1.** Summary of process parameters including oxygen transfer rate (OTR), carbon transfer rate (CTR), the respiratory quotient (RQ) and specific oxygen uptake (qO_2_), glucose (q_glc_), ammonia (q_am_) uptake and carbon dioxide consumption (qCO_2_) rates.

|  | **S_0_** | | | | **S_1_** | | | |
| --- | --- | --- | --- | --- | --- | --- | --- | --- |
|  | **Cultivation 1** | | **Cultivation 2** | | **Cultivation 1** | | **Cultivation 2** | |
|  | **mean** | **s.d.** | **mean** | **s.d.** | **mean** | **s.d.** | **mean** | **s.d.** |
| **OTR (mmol L^-1^ h^-1^)** | 39.154 | 0.058 | 40.804 | 0.264 | 44.254 | 0.924 | 43.740 | 0.204 |
| **CTR (mmol L^-1^ h^-1^)** | 39.437 | 0.288 | 39.624 | 0.141 | 44.128 | 0.695 | 43.316 | 0.367 |
| **q_O2_ (mmol g^-1^ h^-1^)** | 8.471 | 0.089 | 8.479 | 0.037 | 10.080 | 0.479 | 9.092 | 0.154 |
| **q_CO2_ (mmol g^-1^ h^-1^)** | 8.532 | 0.027 | 8.234 | 0.064 | 10.051 | 0.461 | 9.005 | 0.188 |
| **RQ (-)** | 1.007 | - | 0.971 | - | 0.997 | - | 0.990 | - |
| **q_glc_ (g g^-1^ h^-1^)** | 0.599 | 0.003 | 0.578 | 0.005 | 0.658 | 0.022 | 0.591 | 0.011 |
| **q_am_ (g g^-1^ h^-1^)** | 0.045 | 0.000 | 0.041 | 0.000 | 0.047 | 0.001 | 0.041 | 0.001 |

Specific uptake rates were calculated at different time points using the total STR-PFR volume (1.5 L). Values represent the arithmetic mean of samples taken at S_0_ or S_1_ ± s.d. (standard deviation).

**Table S2.** Logarithmic expression ratios of σ^N^-dependent genes/operons involved in the nitrogen regulatory (Ntr) response between sample port PFR P5 and STR S.

| **Gene/ operon** | **Logarithmic ratio^a^ (P5 vs. S) at:** | | | **Function** |
| --- | --- | --- | --- | --- |
|  | **25 min** | **120 min** | **28 h** |  |
| *glnK-amtB* | 1.32/0.66 | 1.34/0.61 | 1.38/0.69 | GlnK – nitrogen regulatory protein, AmtB – ammonia transport |
| *glnHPQ* | 1.02 | 1.01 | 0.98 | Glutamine ABC transporter |
| *glnALG* | 0.74 | 0.86 | 0.78 | GlnA – glutamine synthetase, GlnLG – NtrBC two-component system |
| *nac-cbl* | 1.06 | 1.05 | 1.04 | Nitrogen limitation response adapter for σ^70^ dependent genes |
| *ddpXABCDF* | 1.10 | 1.26 | 1.32 | D-ala D-ala dipeptide transport and dipeptidase |
| *argT* | 0.73 | 0.71 | 0.76 | Lysine/arginine/ornithine ABC transporter |
| *astCADBE* | 1.00 | 0.98 | 0.96 | Arginine catabolic pathway |
| *ssrS* | 0.77 | 0.31 | 0.58 | 6S RNA involved in stationary phase transcription |
| *ycdMLKJIHG* | 0.84 | 0.86 | 0.95 | Pyrimidine degradation |
| *ygjG* | 0.72 | 0.68 | 0.69 | Putrescine degradative pathway |
| *yhdWXYZ* | 0.68 | 0.58 | 0.54 | Polar amino acid transport |
| *yeaGH* | 0.52 | 0.53 | 0.53 | Unknown |

^a^ Underlining indicates significant differential expression. Logarithmic ratios are always given for the first gene in the transcription unit.

**Table S3.** Logarithmic expression ratios of σ^N^ dependent genes/operons not directly involved in nitrogen metabolism between sample port PFR P5 and STR S.

| **Gene/ operon** | **Logarithmic ratio^a^ (P5 vs. S) at:** | | | **Function** |
| --- | --- | --- | --- | --- |
|  | **25 min** | **120 min** | **28 h** |  |
| *chaC* | 0.96 | 0.88 | 0.89 | Predicted glutathione-specific gamma-glutamylcyclotransferase |
| *hycABCDEFGHI* | 0.7 | 1.35 | 1.22 | Formate metabolism – encodes formate hydrogenlyase complex |
| *hypABCDE* | 0.56 | 1.13 | 1.10 | Formate metabolism – encodes proteins for processing hydrogenase 3 |
| *prpBCDE* | 1.01 | 0.69 | 0.72 | Propionate catabolism |
| *rpoH* | 0.69 | 0.54 | 0.64 | Heat shock response sigma factor |
| *rtcBA* | 0.58 | 0.62 | 0.60 | RtcB – RNA splicing ligase, RtcA – 3’-terminal phosphate cyclase |
| *zraP* | 0.93 | 0.79 | 0.80 | Zinc responsive, periplasmic protein with chaperone activity |
| *zraSR* | 0.76 | 0.84 | 0.52 | Two-component system involved in envelope stress response/Pb^2+^ and Zn^2+^ resistance |

^a^ Underlining indicates significant differential expression. Logarithmic ratios are always given for the first gene in the transcription unit.

**Table S4.** Differentially expressed genes (FDR <0.01) with the highest estimated energy demands for transcription and translation.

| ***Gene*^a^** | **Add-on to maintenance^b^, %** | | | | **COG** | | **Function** |  |
| --- | --- | --- | --- | --- | --- | --- | --- | --- |
|  | **mRNA synthesis** | | **TL** | **∑** |  |  | | |
| *glnK* |  | 0.365 | 0.054 | 0.419 | T | Nitrogen regulatory protein PII | | |
| *trpE* |  | 0.230 | 0.033 | 0.263 | E | Anthranilate/para-aminobenzoate synthases component I | | |
| *astC* |  | 0.170 | 0.025 | 0.195 | E | Acetylornithine/succinyldiaminopimelate/putrescine aminotransferase | | |
| *glnH* |  | 0.117 | 0.017 | 0.134 | E | ABC-type amino acid transport/signal transduction system, periplasmic component/domain | | |
| *ynfM* |  | 0.105 | 0.015 | 0.120 | G | Predicted arabinose efflux permease, MFS family | | |
| *uspA* |  | 0.073 | 0.011 | 0.084 | T | Nucleotide-binding universal stress protein, UspA family | | |
| *yfiA* |  | 0.068 | 0.010 | 0.079 | J | Ribosome-associated translation inhibitor RaiA | | |
| *mtr* |  | 0.053 | 0.008 | 0.061 | E | Amino acid permease | | |
| *rpoH* |  | 0.042 | 0.006 | 0.048 | K | DNA-directed RNA polymerase, sigma subunit 32 | | |
| *rseA* |  | 0.042 | 0.006 | 0.048 | T | Negative regulator of sigma E activity | | |
| *osmE* |  | 0.038 | 0.005 | 0.044 | M | Outer membrane protein assembly factor, lipoprotein component | | |
| *hisG* |  | 0.036 | 0.005 | 0.042 | E | ATP phosphoribosyltransferase | | |
| *cpxP* |  | 0.036 | 0.004 | 0.041 | O | Periplasmic protein refolding chaperone Spy/CpxP family | | |
| *cysP* |  | 0.030 | 0.004 | 0.035 | P | ABC-type sulfate transport system, periplasmic component | | |
| *yjbA* |  | 0.028 | 0.004 | 0.032 | R | Phosphate starvation-inducible membrane PsiE (function unknown) | | |
| *yrbL* |  | 0.027 | 0.004 | 0.031 | T | Serine/threonine protein kinase | | |
| *trpL* |  | 0.027 | 0.003 | 0.030 | n.a. | Tryptophan operon leader peptide | | |
| *clpP* |  | 0.022 | 0.003 | 0.025 | O | ATP-dependent protease ClpP, protease subunit | | |
| *smg* |  | 0.019 | 0.002 | 0.021 | S | Uncharacterized conserved protein Smg | | |
| *gst* |  | 0.016 | 0.002 | 0.019 | O | Glutathione S-transferase | | |

**^a^** Genes which expression was significantly changed between STR and PFR P5 (FDR < 0.01) to all sampling times were selected for the calculations (core genes).

^b^ Growth-independent maintenance taken from [Taymaz-Nikerel et al. (2010](#_ENREF_69)).

**Table S5**: List of variants identified after 28 h of cultivation in the STR-PFR system.

| **Gene^1)^** | **Exp./Rep.^2)^** | **Position^3)^** | **Reference^4)^** | **Alternate^4)^** | **Effect** |
| --- | --- | --- | --- | --- | --- |
| *yccE* | Am/1 | 1065700 | C | T | Non-synonymous coding  (Missense\|Cgt/Tgt\|R415C) |
| *paaX* | Am/2 | 1465177 | A | C | Upstream |
| *paaK* | Am/2 | 1465177 | A | C | Downstream |
| *ykgJ* | Am/2 | 303522 | TTACC | CTGCG | Upstream |
| *yagV* | Am/2 | 303522 | TTACC | CTGCG | Downstream |
| *nohA* | Glc/1 | 1638250 | AAACCC | GAATCT | Upstream |
| *yajR* | Glc/3 | 445998 | GAAAAAAG | GAAAAAAAG | Upstream |
| *cyoE* | Glc/3 | 445998 | GAAAAAAG | GAAAAAAAG | Downstream |
| *ydbC* | Glc/2 | 1476826 | CTTTTTTTC | CTTTTTTTTC | Downstream |
| *ydbD* | Glc/2 | 1476826 | CTTTTTTTC | CTTTTTTTTC | Upstream |

^1)^ Gene product functions: *yccE*, putative protein; *paaX*, transcriptional regulation of genes involved in phenylacetic acid catabolism; *paaK*, phenylacetate-CoA ligase; *ykgJ*, predicted ferredoxin; *yagV*, phenylacetate-CoA ligase, *nohA*, Qin prophage - predicted packaging protein; *yajR*, putative transport protein, major facilitator superfamily; *cyoE*, heme O synthase of the cytochrome *bo* complex; *ydbC*, peptide transporter induced by carbon starvation; *ydbD*, conserved protein.

^2)^ Exp./Rep. refers to the experimental conditions either glucose (Glc) or ammonia (Am) limitation/starvation and number of biological replicate, respectively.

^3)^ Position refers to the position of the first base in the string.

^4)^ Reference and alternate base(s).

# Supplementary Methods

## **Methods S1. Plug flow reactor characterization**

Prior to culture testing, the STR-PFR two-compartment system was characterized regarding plug-flow behavior and mean residence times of cells in the STR and PFR, respectively. Additionally, residence times were determined for each sample port along the PFR. During PFR characterization conductivity probes were installed at every sample port, while distilled water was pumped through the system to establish typical cultivation conditions using the same pump flow rate, aeration (in the PFR) and overpressure. Then, a tracer consisting of 3 mL of K_2_HPO_4_ (3 M) was pulsed (Buchholz *et al.*, 2014) and conductometers (LF 521, WTW Wissenschaftlich-Technische Werkstätten GmbH, Weilheim, Germany) were used to record response curves (Supplementary Fig. S9). According to Levenspiel (2012), the average residence time $\tau$ and its variance $\sigma^{2}$ (in min) were calculated for each sample port ($\tau_{P1}-\tau_{P5}$) and the complete PFR ($\tau_{PFR})$ :

| $\tau=\frac{\sum_{i}^{n-i} t_{i}\cdot c_{i}\cdot\Delta t_{i}}{\sum_{i}^{n-i} c_{i}\cdot\Delta t_{i}}$ | (1) |
| --- | --- |
| $\sigma^{2}=\frac{\sum_{i}^{n-i} t_{i}^{2}\cdot c_{i}\cdot\Delta t_{i}}{\sum_{i}^{n-i} c_{i}\cdot\Delta t_{i}}-\tau^{2}$ | (2) |

where the conductivity signal $c$ corresponds to the tracer concentration at the time point after induction of the tracer pulse$t$ at the measuring interval $i$. The Bodenstein number $Bo$ was calculated from the mean residence time and its variance as defined by George *et al.*, (1993) and Levenspiel (1999), to characterize back-mixing effects in the PFR:

| $Bo=\frac{2{\tau^{2}}}{\sigma_{\tau}^{2}}$ | (3) |
| --- | --- |

A $Bo$ > 10 is generally considered to be approaching plug-flow behavior so that back-mixing effects can be excluded (George *et al.*, 1993).

## **Methods S2. Gene grouping for the comparison of ammonia and glucose transcriptome data**

Short- and long-term transcriptional responses conducted after 28 h were compared under periodic glucose and ammonia shortage; represented by the contrasts P5 versus S and S versus S_0_, respectively. In this context, the absolute differential expression ratio between the two nutritional conditions (|log_2_ (Δ_N_)| – |log_2_ (Δ_C_)|) was calculated for each contrast and following this genes were assigned into two groups based on their distribution, where Δ_N_ and Δ_C_ indicate the logarithmic expression ratio under ammonia or glucose shortage, respectively (see Supplementary Fig. S10).

Group A includes genes, for which the change in gene expression to short- or long-term stress imposed by the PFR was comparable under the two nutrient-starved conditions, i.e. |Δ_N_| ≈ |Δ_C_|. For gene selection, one standard deviation from the mean was chosen as maximum relative difference allowed between the expression ratios. The group could be further divided into genes that are regulated in the same direction and genes regulated in opposing directions under the nutrient conditions. Since for the majority of genes the differential expression between both nutrient conditions was maintained, only genes that were significantly differentially expressed at short- or long-term in at least one dataset were considered in group A (gene lists are included in Supplementary Information).

Group B comprises genes for which Δ_N_ is markedly different from Δ_C_ fulfilling a minimal difference of two standard deviations from the mean (Supplementary Fig. S10) and was further separated into two subgroups, i.e. genes whose absolute logarithmic expression ratios were always higher under ammonia than under glucose shortage (|Δ_N_| >> |Δ_C_|) and vice versa (|Δ_N_| << |Δ_C_|). Again only genes that were significantly differentially expressed at short- or long-term in at least one dataset were selected for group B. Therefore, genes within these subgroups showed strong transcriptional differences (induction or repression) to repeated short-term starvation between the two nutrient cultivations (Supplementary Information).

In addition to nutrient-specific genes for which high absolute differences in expression ratio existed, 249 and 49 genes significantly differentially expressed at P5 and S under either ammonia or glucose conditions could not be clearly categorized into one of the subgroups defined by the selection criteria shown in Supplementary Fig. S10 (+1σ ≤ δ < +2σ or -1σ ≤ δ < -2σ). These genes are also presented in Supplementary Information, but were excluded from Fig. 5 in the main manuscript.

## **Methods S3. Estimation of ATP cost of gene expression**

The overall ATP requirements for transcription between PFR P5 and STR were estimated by balancing the individual nucleotide triphosphates (NTPs) required for mRNA synthesis and the nucleotide monophosphates (NMPs) produced by mRNA degradation along the PFR. For this reason, transcripts that were significantly changed (FDR <0.01) under ammonia shortage were selected for each time point and divided into up- and downregulated gene sets. The individual protein-coding nucleotide sequences from RegulonDB v. 8.0 (Salgado *et al.*, 2013) were used to compute the nucleotide fractions for each gene in these sets. Next, TPM/10^6^ were computed and multiplied by the respective fraction of A, C, G, U nucleotides, leading to the proportion of nucleotides of the transcriptome for a given gene (Li *et al.*, 2010). Based on this proportion, the number of molecules of each nucleotide was determined and balanced as described in detail in Löffler, Simen *et al.,* (2016). In this context, costs for NMP recycling were assumed with 2 ATP per nucleotide, whereas individual ATP costs were considered for *de novo* NTP synthesis. For computation of translation costs, the number of amino acid molecules required for one mRNA translation was estimated from the number of NTP molecules required for mRNA synthesis along the PFR. It was assumed that a nucleotide triplet is needed for each amino acid and that after mRNA occurrence, 11 proteins were translated per mRNA (estimated for µ=0.2 h^-1^ from Bremer and Dennis (1996)). Translation costs are 4 ATP per amino acid (Stouthamer, 1973; Kaleta *et al.*, 2013). By multiplying the combined costs with the number of required amino acid molecules per translation, the overall ATP cost was calculated. To obtain the overall ATP cost, computation of nucleotide and amino acid costs from precursors was performed as described in Löffler, Simen *et al.* (2006) and is based on work of Kaleta *et al.* (2013). The key factors that lead to the additional ATP requirements for transcription and translation are presented in Supplementary Fig. S11; the values indicate the percent increase in ATP with respect to growth-independent maintenance of 0.0027 mol of ATP g (DW)^-1^ h^-1^ given by Taymaz-Nikerel *et al.* (2010).

## **Methods S4: Genetic variant discovery**

Variant calling was performed on the sets of reads aligned to the NCBI reference genome of *E. coli* W3110 (NC_007779) using FreeBayes (Garrison and Marth, 2012) (https://github.com/ekg/freebayes) and SNPs were annotated using SNPEff (Cingolani *et al.*, 2012) (http://snpeff.sourceforge.net/). For the called variants the following cutoffs were applied: per-base quality score >20 (i.e., error rate in base calling <1%) and coverage per called SNP >3 reads. In addition, at least 80% of total reads should contain a variant at this position, to reduce the number of false positive hits. The 5’ and 3’ untranslated regions (UTR) were set at 200 bases for all genes, since the length of most 5’ and 3’ UTRs is ≤200 bases as determined from the distribution of *E. coli* K12 UTRs derived from the RegulonDB database (Salgado et al., 2013).

The analysis revealed that very few variants were identified after 25, 26 or 28 h in the cycling system (Supplementary Table S5). The majority of variants were found up or downstream of the coding region. Due to the fixed setting of 200 bases, variants detected in 5’ and 3’ UTRs of several genes do overlap. Closer examination of the up- and downstream sequences of these genes revealed that in all cases the actual length of 5’ UTRs annotated in RegulonDB v. 8 (Salgado et al., 2013) was much shorter than the 200 bases estimated. In addition, no 3’ UTRs were annotated for the detected genes. Consequently, the coding and regulatory regions of these genes should not be affected by the variants. One non-synonymous coding variant was identified leading to an amino acid exchange (R415C) in *yccE,* which encodes a putative protein. However, read counts found for *yccE* were very low in all samples so that it was excluded from statistical analysis according to the non-specific filtering criteria (for details see Materials and methods section). In addition, none of the variants was found in more than one biological replicate suggesting no systematic selection triggered by the cycling system. Thus, we assume that under the present conditions rapid on/off switching of genes may be sufficient to cope with the applied stress.

# References

Bremer, H., and Dennis, P.P. (1996) Modulation of Chemical Composition and Other Parameters of the Cell by Growth Rate. In *Escherichia coli and Salmonella*. Neidhart, F.C. (ed.). ASM Press, Washington D.C. pp. 1553–1569.

Buchholz, J., Graf, M., Freund, A., Busche, T., Kalinowski, J., Blombach, B., and Takors, R. (2014) CO_2_ /HCO_3_^−^ perturbations of simulated large scale gradients in a scale-down device cause fast transcriptional responses in *Corynebacterium glutamicum*. *Appl Microbiol Biotechnol* **98**: 8563–8572.

Chilcott, G.S., and Hughes, K.T. (2000) Coupling of flagellar gene expression to flagellar assembly in *Salmonella enterica* Serovar Typhimurium and *Escherichia coli*. *Microbiol Mol Biol Rev* **64**: 694–708.

Cingolani, P., Platts, A., Wang, L.L., Coon, M., Nguyen, T., Wang, L., *et al.* (2012) A program for annotating and predicting the effects of single nucleotide polymorphisms, SnpEff: SNPs in the genome of Drosophila melanogaster strain w 1118; iso-2; iso-3. *Fly (Austin)* **6**: 80–92.

Garrison, E., and Marth, G. (2012) Haplotype-based variant detection from short-read sequencing. *arXiv Prepr arXiv12073907 [q-bioGN]* 9.

George, S., Larsson, G., and Enfors, S.O. (1993) A scale-down two-compartment reactor with controlled substrate oscillations: Metabolic response of *Saccharomyces cerevisiae*. *Bioprocess Eng* **9**: 249–257.

Kaleta, C., Schäuble, S., Rinas, U., and Schuster, S. (2013) Metabolic costs of amino acid and protein production in *Escherichia coli*. *Biotechnol J* **8**: 1105–1114.

Levenspiel, O. (1999) *Chemical reaction engineering*. 3rd ed., John Wiley & Sons, Ltd, New York, New York, USA.

Levenspiel, O. (2012) *Tracer Technology: Modeling the Flow of Fluids (Fluid Mechanics and its Applications)*. Springer Open Ltd, New York, New York, USA.

Li, B., Ruotti, V., Stewart, R.M., Thomson, J. a, and Dewey, C.N. (2010) RNA-Seq gene expression estimation with read mapping uncertainty. *Bioinformatics* **26**: 493–500.

Löffler, M., Simen, J.D., Jäger, G., Schäferhoff, K., Freund, A., and Takors, R. (2016) Engineering *E. coli* for Large-Scale Production - Strategies Considering ATP Expenses and Transcriptional Responses. *Metab Eng* **38**: 73–85.

Luo, W., Friedman, M.S., Shedden, K., Hankenson, K.D., and Woolf, P.J. (2009) GAGE: generally applicable gene set enrichment for pathway analysis. *BMC Bioinformatics* **10**: 161.

Salgado, H., Peralta-Gil, M., Gama-Castro, S., Santos-Zavaleta, A., Muñiz-Rascado, L., García-Sotelo, J.S., *et al.* (2013) RegulonDB v8.0: omics data sets, evolutionary conservation, regulatory phrases, cross-validated gold standards and more. *Nucleic Acids Res* **41**: D203–D213.

Stouthamer, A.H. (1973) A theoretical study on the amount of ATP required for synthesis of microbial cell material. *Antonie Van Leeuwenhoek* **39**: 545–565.

Tatusov, R.L., Galperin, M.Y., Natale, D. a, and Koonin, E. V (2000) The COG database: a tool for genome-scale analysis of protein functions and evolution. *Nucleic Acids Res* **28**: 33–36.

Taymaz-Nikerel, H., Borujeni, A.E., Verheijen, P.J.T., Heijnen, J.J., and Gulik, W.M. van (2010) Genome-derived minimal metabolic models for *Escherichia coli* MG1655 with estimated in vivo respiratory ATP stoichiometry. *Biotechnol Bioeng* **107**: 369–381.

Traxler, M.F., Zacharia, V.M., Marquardt, S., Summers, S.M., Nguyen, H.T., Stark, S.E., and Conway, T. (2011) Discretely calibrated regulatory loops controlled by ppGpp partition gene induction across the “feast to famine” gradient in *Escherichia coli*. *Mol Microbiol* **79**: 830–845.
